# Health related quality of life is differently associated with leisure-time physical activity intensities according to gender: a cross-sectional approach

**DOI:** 10.1186/1477-7525-12-98

**Published:** 2014-08-18

**Authors:** Priscila Missaki Nakamura, Inaian Pignatti Teixeira, Bruno Paula Caraça Smirmaul, Emerson Sebastião, Camila Bosquiero Papini, Sebastião Gobbi, Eduardo Kokubun

**Affiliations:** Department of Physical Education, Physical Activity, Health and Sport Laboratory (NAFES)–São Paulo State University, Rio Claro, SP Brazil; Aging and Diversity Lab. Department of Kinesiology and Community Health, University of Illinois at Urbana-Champaign, Champaign, USA; Department of Physical Education, Physical Activity and Aging Lab. Univ. São Paulo State University, Rio Claro, Brazil; Departamento de Educação Física, Universidade Estadual Paulista. Instituto de Biociências, Av. 24-A. n 1515. Bela Vista, Rio Claro, SP CEP 13506-900 Brazil

**Keywords:** Health-related quality of life, Adult, Intensities of physical activity, Brazil, Mental health, Physical health, Qualidade de vida relacionada à saúde, Adultos, Intensidade de atividade física, Brasil, Saúde física, Saúde mental

## Abstract

**Background:**

Several studies have demonstrated a positive association between physical activity (PA) and health-related quality of life (HRQL). However, studies have suggested that this association depends both on the PA intensity and the domain of HRQL evaluated. This study aimed to explore the association between physical, mental and overall HRQL with recommended levels of PA. PA levels were divided into moderate and vigorous intensity leisure-time PA and total leisure-time PA.

**Methods:**

The study included 1001 adults, 582 women (46 ± 17 years) and 419 men (43 ± 16 years), residents in Rio Claro-SP, Brazil. All participants completed the SF-36 questionnaire to assess HRQL and the long version of the International Physical Activity Questionnaire (IPAQ) to assess level and intensities of leisure-time PA. Total leisure-time PA at moderate intensity was classified as: less than 9 min/week, 10-149 min/week, 150-299 min/week and 300 min/week or more. Total leisure-time PA at vigorous intensity was classified as: less than 9 min/week, 10 to 74.9 min/week, 75-149 min/week and 150 min/week or more. Multiple linear regression was performed in STATA version 12.0.

**Results:**

Among women, moderate intensity and total leisure-time PA were associated with physical health. Among men, moderate and vigorous intensity and total leisure-time PA were associated with physical health and overall HRQL. Furthermore, moderate intensity and total leisure-time PA were associated with mental health in men. However, vigorous intensity PA was not associated with mental health for this group.

**Conclusion:**

The different domains of HRQL were associated with different levels and intensities of PA in leisure-time according to gender of adults. These findings indicate the complexity and importance of evaluating the HRQL stratified by gender and consider the different levels and intensities of PA.

## Resumo

**Introdução:**

Diversos estudos demonstram associação positiva entre prática de atividade física (AF) e qualidade de vida relacionada à saúde (QDV). Entretanto, estudos sugerem que essa influência dependa tanto da intensidade da AF, bem como do domínio da QDV avaliado.

**Objetivo:**

Explorar à associação entre os domínios físico, mental e a QDV total com os níveis de AF recomendados, divididos nas intensidades moderada e vigorosa e AF total no lazer em adultos.

**Metodologia:**

Participaram do presente estudo 1001 adultos, 582 mulheres (46 ± 17 anos) e 419 homens (43 ± 16 anos) residentes da cidade de Rio Claro-SP. Todos os participantes responderam o questionário SF-36 para avaliar a QDV e o International Physical Activity Questionnarie (IPAQ) - Long Version para avaliar o nível de AF no lazer. A AF total no lazer e de intensidade moderada foi classificada em menor que 9 min/semana, 10 a 149 min/semana, 150 a 299 min/semana e acima de 300 min/semana. A AF vigorosa foi classificada em menor que 9 min/semana, 10-74.9 min/semana, 75 a 149 min/semana e acima de 150 min/semana. Foi realizado a Análise de regressão linear múltipla no STATA versão 12.0.

**Resultados:**

Entre as mulheres, apenas o domínio de saúde física associou-se com os níveis de AF moderada e total do lazer. Já para os homens, o domínio de saúde física e a QDV total foram associados com o nível de AF total no lazer, atividade moderada e vigorosa. Ainda entre os homens, no domínio de saúde mental foi associado com AF de intensidade moderada e AF total no lazer, porém não foi associado com a intensidade vigorosa.

**Conclusão:**

Os diferentes domínios da QDV foram associados com os diferentes níveis e intensidades de AF realizadas no momento do lazer de acordo com o gênero em adultos. Esses achados indicam a complexidade e a importância de se avaliar a QVD estratificado por gênero e considerar diferentes níveis e intensidades de AF.

## Background

Quality of life is considered to be a complex construct that is subjective and dynamic. Nahas [1] stated that health-related quality of life (HRQL) is altered by a combination of multiple factors that influence the daily life of human beings. Such factors can be classified as environmental (housing, transportation, security, education, compensation and leisure activities) or individual (heredity and lifestyle). The construct of HRQL can be studied in different ways. HRQL, for example, is a condition that goes beyond the absence of disease, and overcoming the difficulties related to morbidity [2, 3]. Additionally, it refers not only to how people perceive their general health, but also to specific states of physical, psychological and social support that is provided to carry out activities of daily life [4]. Evaluating HRQL enables us to investigate its influencing factors and consequently, create interventions to improve it, especially relieving pain, malaise and consequences of diseases [3].

Cross-sectional studies have shown a consistent and positive association between physical activity (PA) and both physical and functional domains of HRQL (such as the psychological and emotional aspects). This association was found to be true for individuals with chronic non-communicable diseases [5] and healthy individuals [6–8]. However, most studies in the literature have evaluated the influence of leisure-time PA [8–10] on HRQL, not considering the specific variations regarding level and intensities of PA as well as the distinct physical and mental HRQL domains [11]. The benefits of regular moderate or vigorous PA adoption have been recommended for rehabilitation, maintenance and promotion of physical and mental health in different populations [11]. However, the dose-response relationship between PA and HRQL remains controversial. While lower level of PA than the recommended is associated with lower HRQL [12], PA performed above the recommended levels has been associated with lower [13] and higher [14] HRQL. Guimarães et al. [15] found that each HRQL domain relates differently depending on PA intensity. However, this study comprised only middle-aged women and PA recommendation was not considered according to intensities.

Thereby, the aim of this study was to investigate the association between physical, mental and overall HRQL with the recommended levels of PA, divided into moderate and vigorous intensities, as well as total leisure-time PA in adults of a medium sized city. Furthermore, as suggested by Brown et al. [13], we also investigated the dose-response relationship between leisure-time PA and HRQL. We hypothesize that higher levels of PA at moderate and vigorous intensities, as well as total leisure-time PA, will be associated with higher scores for the HRQL domains assessed.

## Methods

cross-sectional population-based study was conducted in the city of Rio Claro. Rio Claro is located 180 kilometres far from the capital Sao Paulo, Brazil. The city presents a land area of approximately 1498 km^2^; a population density of 373.47 (habitants/km^2^), a total population of 187.637 [16], and a Human Development Index of 0.825 [17]. The target population of this study were adults aged 20 years or more, living in the urban area of Rio Claro for more than a year.

Adult residents were randomly sampled from stratified census tracts. The city contains 200 census tracts in total and 100 census tracts were selected for this study. Next, eight houses were randomly chosen, each sector totalling 800 houses to be included in the study. All residents, 20 years of age or older, who did not have severe motor disabilities (e.g. quadriplegic, cerebral palsy, etc.) were interviewed.

In total, 1464 households were randomly selected. Of these, only 66% (960) were eligible for the study and the others had enrolment problems. The total number of households interviewed was 800 (83%), and approximately 1700 individuals aged 20 years or older were part of the data collection, with 17% of refusals (individuals who did not respond the questionnaire; reported lack of time; were not found in five attempts by the interviewers; presented health problems).

A pilot run using a census tract that was not used in the main study was carried out to test the data collection instruments, in order to standardize the collection procedures. Following, we selected ten individuals, both male and female, with a high school diploma as interviewers. They received 40-hours of training, encompassing theoretical study of interviewing techniques, role and application of the questionnaire using a manual developed specifically for this purpose. Two clerks of both genders who had a high school degree and basic knowledge of the database (EPI INFO) were also hired.

This study was approved by the Ethics Committee of the São Paulo State University- Rio Claro-SP (No. 0848) and each participant received information on the consent form prior to data collection.

### Questionnaires

The questionnaires were administered individually with an average time of 30 minutes each. The quality control was carried out by revisiting 10% of households. Refusals were when the resident refused to answer the questionnaire or after five visits (three by the interviewers and two by the researchers).

### Health-related quality of life

HRQL was assessed using the SF-36 (Medical Outcomes Study 36 - Item Short Form Health Survey) [18], translated and validated to Brazilian Portuguese [19]. The SF-36 consists of 36 items grouped into eight main components that constitute the three main domains: physical health, mental health and overall HRQL. The physical health section encompasses physical components of physical functioning, role physical, bodily pain and general health. The field of mental health encompasses the vitality, social functioning, and emotional and mental health. For HRQL a total score is generated with the average scores of the eight components. The three domains present a final score ranging from 0 to 100, representing the worst and best health status possible, respectively.

### Level of PA during leisure-time

To measure the level of total leisure-time PA, as well as PA intensity (vigorous and moderate), the International Physical Activity Questionnaire (IPAQ) - long version, translated and validated for the Brazilian Portuguese [20] was used. The questions used were related to total leisure-time PA performed during the last week before the interview with a minimum duration of 10 minutes per session. The leisure-time PA of moderate intensity was classified as: less than 9 min/week (level 1), 10-149 min/week (level 2), 150-299 min/week (level 3) and 300 min/week or more (level 4) (twice the recommended levels at moderate intensity). Vigorous PA was classified as: less than 9 min/week (level 1), 10 to 74.9 min/week (level 2), 75-149 min/week (level 3) and 150 min/week or more (level 4) (twice the recommended levels at vigorous intensity).

### Gender, age, education and socioeconomic status

Individual information regarding gender, age, educational level and socioeconomic status were also assessed. The educational level was assessed by the question “What was your final year of study?”. Response options were: a) none or incomplete primary; b) primary complete or junior high incomplete; c) junior high complete or high school incomplete; d) high school complete or university incomplete an e) university complete.

The socioeconomic status was assessed by a questionnaire developed by the Brazilian Research Association [21], establishing economic classification criterion based on the estimate of the purchasing power of people and urban families.

### Statistical analysis

After testing for data normality using the Kolmogorov-Smirnov test, the results were expressed as means ± SD. The first analysis to verify the association between HRQL domains and total leisure-time PA at moderate and vigorous intensities was the linear regression (r^2^). After that, the multiple linear regression stratified by gender was used. The variables age, education and socioeconomic status were used as control variables, being categorized as follows: age (20-39 years, 40-59 years and ≥ 60 years), educational level (≤8 years, 9-11 years, ≥ 12 years); socioeconomic status (high, medium and low). All analyses were performed using Stata version 12.0.

## Results

The study sample contains 1001 participants, being mostly women (58.1%), people aged 20-39 years (41.3%), socioeconomic status medium (44.1%), education level ≥ 12 (46.4) total leisure-time PA less than 9 min/week (59.7%), moderate PA level less than 9 min/week (63.3%) and vigorous PA less than 9 min/week (88.9%) (Table 1).

**Table 1:** **Sample characteristics (n = 1001, Rio Claro-SP)**

Variable	Total n (%)	Women n (%)	Men n (%)
**Gender**			
Women	582 (58.1)	-	-
Men	419 (41.8)	-	-
**Age group (years)**			
20-39	414 (41.3)	232 (39.8)	182 (43.4)
40-59	370 (36.9)	213 (36.6)	157 (37.4)
≥ 60	217 (21.6)	137 (23.5)	80 (19.0)
Socioeconomic status*			
High	366 (37.3)	196 (34.7)	170 (41.0)
Medium	432 (44.1)	253 (44.9)	179 (43.1)
Low	181 (18.4)	115 (20.4)	66 (15.9)
**Educational level (years) ¥**			
≤ 8	353 (35.3)	234 (40.3)	119 (28.4)
9-11	183 (18.3)	113 (19.4)	70 (16.7)
≥ 12	464 (46.4)	234 (40.3)	230 (54.9)
**Level of leisure-time of PA (min/week)**			
≤ 9	598 (59.7)	378 (64.9)	220 (52.5)
10-149	113 (11.3)	53 (9.1)	60 (14.3)
150-299	115 (11.5)	65 (11.2)	50 (11.9)
≥ 300	175 (17.5)	86 (14.8)	89 (21.3)
**Moderate PA (min/week)**			
≤ 9	634 (63.3)	386 (66.3)	248 (59.2)
10-149	133 (13.3)	61 (10.5)	72 (17.2)
150-299	121 (12.1)	69 (11.9)	52 (12.4)
≥ 300	113 (11.3)	66 (11.3)	47 (11.2)
**Vigorous PA (min/week)**			
≤ 9	890 (88.9)	540 (92.8)	350 (83.5)
10-74.9	18 (1.8)	6 (1.0)	12 (2.9)
75-149	26 (2.6)	10 (1.7)	16 (3.8)
≥ 150	67 (6.7)	26 (4.5)	41 (9.8)

*979 participants.

¥1000 participants.

**Figure 1:**
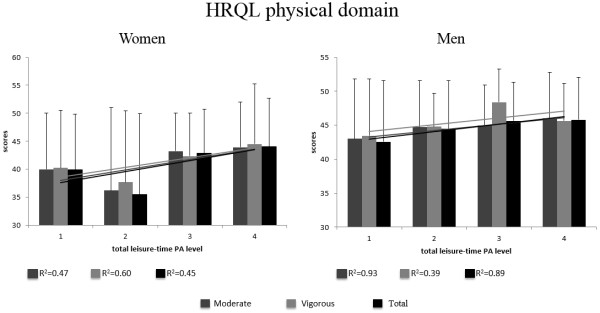
**Average scores of the HRQL physical domain stratified by gender and level and intensity of leisure-time PA.**

Figure 1 presents the average scores of the HRQL physical and the coefficient of determination (r^2^) of linear regression domain stratified by gender and leisure-time PA (moderate, vigorous and total). The lowest r^2^ value for women and men was 0.45 to total PA and 0.60 to vigorous PA and the highest value was 0.39 to vigorous PA and 0.93 to moderate PA, respectively.

For women, the highest average (44.5) was for vigorous PA at level 4 (150 min/week or more), and for men (48.0) for the vigorous PA at level 3 (75-149 min/week). The lowest scores were for the total leisure-time PA level 2 (9-149 min/week) for women (35.4), and at level 1 (less than 9 min/week) for men (42.5).

**Figure 2:**
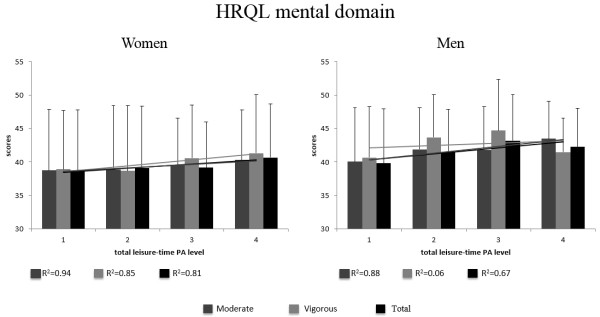
**Average scores for the HRQL mental domain stratified by gender and level and intensity of leisure-time PA.**

Figure 2 shows the average scores for the HRQL mental domain and the coefficient of determination (r^2^) of linear regression stratified by gender and leisure-time PA (moderate, vigorous and total). The lowest r^2^ value for women and men was 0.81 to moderate PA and 0.06 to vigorous PA and the highest value was 0.94 to vigorous PA and 0.88 to moderate PA, respectively.

For women, the highest average of HRQL mental domain (41.3) was for vigorous activity at level 4 (150 min/week or more) and the lowest (38.6) was for vigorous intensity PA at level 2 (from 10 to 74.9 min/week). For men, the highest average (43.4) was for moderate activity level 4 (300 min/week or more) and the lowest (39.8) was for leisure-time total PA at level 1 (less than 9 min/week).

**Figure 3:**
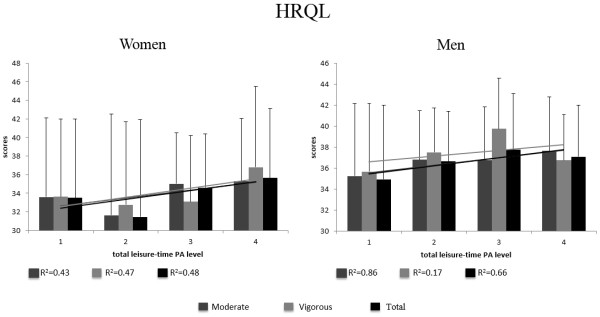
**Average scores of overall HRQL stratified gender and level of leisure-time PA.**

Figure 3 shows the average scores for the overall HRQL and coefficient of determination (r^2^) of linear regression, stratified by gender and level and intensities of leisure-time PA (moderate, vigorous and total). The lowest r^2^ value for women and men was 0.43 to moderate PA and 0.17 to vigorous PA, respectively. The highest value of r^2^ was 0.48 to total PA and 0.86 to moderate PA, from women and men respectively.

For women, the highest average (36.7) was for vigorous intensity level 4 (150 min/week or more) and the lowest (31.4) was for total leisure-time PA at level 2 (9-149 min/week). For men, the highest average (39.7) was for vigorous intensity level 3 (75-149 min/week) and the lowest (34.9) was for total leisure-time PA at level 1 (less than 9 min/week).

The physical health domain for women was associated with the level of moderate leisure-time PA. Related to the men, moderate and total leisure-time PA were associated with physical health. In the adjusted analysis, women who had between 10 and 149 min/week of total leisure-time PA registered a score 4.3 points lower compared with inactive women. However, women who were physically active for 300 min/week or more presented 2.8 points higher than those who had done less than 9 min/week. Additionally, the same behaviour was observed for women for the moderate intensity. Women who practiced between 10-149 min/week showed 3.6 points less than women who did not engage in any activity, and those who performed more than 300 min/week had 3 points higher than those who had done less than 9 min/week of moderate activity in leisure-time.

For men, the adjusted analysis showed that those who performed 150-299 min/week and 300 min/week or more of total leisure-time PA, presented 2.5 and 2.6 points higher than men who performed less than 9 min/week, respectively. In addition, men who reported 300 min/week or more of moderate activity and between 75-149 min/week of vigorous activity had 2.6 and 4.1 more points than men who performed less than 9 min/week of these activities (Table 2).

**Table 2:** **Crude and adjusted analysis of the physical health domain stratified by gender (n = 1001, Rio Claro-SP)**

	Women				Men			
Variables	Crude		Adjusted*		Crude		Adjusted*	
	β	p	β	p	β	p	β	p
**Level of total leisure-time PA (min/week)**								
≤ 9	1		1		1		1	
10-149	-4.3	0.003	-3.9	0.007	1.9	0.10	1.4	0.23
150-299	2.9	0.03	2.4	0.07	3.0	0.01	2.5	0.04
≥ 300	4.1	0.001	2.8	0.02	3.2	0.001	2.6	0.01
R^2^	0.04		0.14		0.03		0.05	
Adjusted R^2^	0.04		0.13		0.02		0.03	
**Level of moderate PA (min/week)**								
≤ 9	1		1		1		1	
10-149	-3.7	0.007	-3.6	0.008	1.6	0.11	1.01	0.35
150-299	3.2	0.01	2.6	0.04	2.0	0.10	1.6	0.19
≥ 300	3.9	0.004	3.0	0.02	3.0	0.01	2.6	0.04
R^2^	0.04		0.14		0.01		0.05	
Adjusted R^2^	0.03		0.13		0.01		0.03	
**Level of vigorous PA (min/week)**								
≤ 9	1		1		1		1	
10-74.9	-2.5	0.55	-4.8	0.23	1.3	0.57	0.66	0.78
75-149	2.1	0.52	1.9	0.54	4.9	0.01	4.14	0.04
≥ 150	4.2	0.04	0.8	0.68	2.1	0.09	1.31	0.33
R^2^	0.008		0.12		0.012		0.05	
Adjusted R^2^	0.003		0.10		0.010		0.03	

*Age, education level and socioeconomic status.

For women, the mental health domain was not associated with the level of PA, but for men an association was observed with moderate and total leisure-time PA. In the adjusted analysis, men who performed 150 min/week or more of leisure-time PA had higher overall scores in this domain when compared with men who performed less than 9 min/week of PA. Additionally, men who took 300 min/week or more of moderate PA had 3.2 points more than men who performed less than 9 min/week of moderate activity (Table 3).

**Table 3:** **Crude and adjusted analysis of the mental health domain stratified by gender (n=1001, Rio Claro-SP)**

	Women				Men			
Variables	Crude		Adjusted*		Crude		Adjusted*	
	β	p	β	p	β	p	β	p
**Level of total leisure-time PA (min/week)**								
≤ 9	1		1		1		1	
10-149	0.4	0.73	0.3	0.79	1.6	0.12	1.6	0.14
150-299	0.5	0.65	0.1	0.93	3.3	0.00	3.1	0.01
≥ 300	2.0	0.05	1.1	0.28	2.4	0.01	2.2	0.02
R^2^	0.01		0.03		0.03		0.03	
Adjusted R^2^	0.00		0.02		0.02		0.02	
**Level of moderate PA (min/week)**								
≤ 9	1		1		1		1	
10-149	0.1	0.90	-0.0	0.97	1.6	0.12	1.7	0.09
150-299	0.8	0.47	0.3	0.77	3.3	0.00	1.4	0.20
≥ 300	1.5	0.18	0.8	0.49	2.4	0.01	3.2	0.01
R^2^	0.00		0.03		0.03		0.03	
Adjusted R^2^	0.00		0.01		0.02		0.01	
**Level of vigorous PA (min/week)**								
≤ 9	1		1		1		1	
10-74.9	-0.2	0.95	-1.5	0.67	3.0	0.16	2.8	0.20
75-149	1.6	0.55	1.2	0.66	4.0	0.03	3.4	0.07
≥ 150	2.4	0.17	0.9	0.60	0.8	0.49	0.5	0.69
R^2^	0.00		0.03		0.01		0.03	
Adjusted R^2^	0.00		0.01		0.01		0.00	

*Age, education level and socioeconomic status.

Total HRQL for women was not associated with PA, whereas for men it was associated both for moderate and vigorous intensities.

Men performing leisure-time PA for 150-299 min/week or 300 min/week or more had 2.5 and 1.8 points higher than men who performed less than 9 min/week, respectively. Additionally, men who performed 300 min/week or more of moderate PA and between 75-149 min/week of vigorous activity had 2.1 and 3.4 points more for overall HRQL compared with men who performed less than 9 min/week (Table 4).

**Table 4:** **Crude and adjusted analysis of the overall HRQL stratified by gender (n=1001, Rio Claro-SP)**

	Women				Men			
Variables	Crude		Adjusted*		Crude		Adjusted *	
	β	p	β	p	β	p	β	p
**Level of total leisure-time PA (min/week)**								
≤ 9	1		1		1		1	
10-149	-2.03	0.09	-1.76	0.15	1.72	0.06	1.15	0.10
150-299	1.09	0.32	0.70	0.53	2.83	0.003	2.53	0.01
≥ 300	2.15	0.03	1.23	0.22	2.16	0.005	1.81	0.02
R^2^	0.02		0.07		0.03		0.04	
Adjusted R^2^	0.01		0.05		0.02		0.02	
**Level of moderate PA (min/week)**								
≤ 9	1		1		1		1	
10-149	-1.9	0.09	-1.8	0.10	1.5	0.06	1.2	0.15
150-299	1.4	0.19	0.9	0.38	1.5	0.18	1.3	0.17
≥ 300	1.7	0.12	1.0	0.34	2.3	0.01	2.1	0.03
R^2^	0.01		0.07		0.02		0.04	
Adjusted R^2^	0.01		0.05		0.01		0.01	
**Level of vigorous PA (min/week)**								
≤ 9	1		1		1		1	
10-74.9	-0.85	0.80	-2.3	0.49	1.8	0.30	1.4	0.42
75-149	-0.55	0.83	-0.7	0.78	4.0	0.010	3.4	0.03
≥ 150	3.1	0.06	1.2	0.48	1.0	0.29	0.5	0.60
R^2^	0.01		0.07		0.02		0.07	
Adjusted R^2^	0.00		0.05		0.01		0.01	

*Age, education level and socioeconomic status.

## Discussion

The aim of this study was to investigate the association between HRQL (physical, mental health and overall) with leisure-time PA (moderate and vigorous intensities and total) using the PA recommendations as a guideline among the adult population of a medium sized city.

The main findings of this study show that the level and intensities of PA were associated with HRQL. However, the direction and magnitude of this association varies according to the HRQL domain and gender. Furthermore, it was observed that, in general, the recommended levels of PA in both moderate and vigorous intensities are associated with higher HRQL scores.

Among women, only the physical health domain was associated with moderate and total leisure-time PA. For men, the physical health and overall domains were associated with the level of total leisure-time PA, as well as moderate and vigorous PA intensities. There was no significant association between mental health and vigorous intensity PA in men. However, there was an association with moderate intensity and total leisure-time PA.

Women with a physical health domain score ranging from 36 to 44 points performed more than 300 min/week of leisure-time PA, demonstrating that they have a better perception of physical health than inactive women. These results are in agreement with the findings of Olson et al. [22] and Valenti et al. [23], who found that a higher level of leisure-time PA is associated with higher scores on the physical health domain. However, it was found that women who perform 10-149 min/week have 4.3 points less than women who do not conduct any activity. This may have occurred because of the perception of HRQL is “all or nothing” (i.e., women who do not reach the recommended 150 minutes of PA did not perceive HRQL improvements). According to Guimarães et al. [15] and Fox et al. [24], women who perform more than 300 min/week of moderate PA had higher scores in this domain than women who did not perform this activity. Morimoto et al. [25] found that women who underwent vigorous PA (9 METS) had higher physical health scores, an association that was not found in the present study. This difference may be attributed to the different instruments and categorizations of intensity to assess the PA levels.

Men who perform more than 150 min/week of leisure- time PA had higher scores in the physical health domain than men who do not perform this activity. This result is in agreement with the results of Ko et al. [26], Yasunaga et al. [27] and Wendel-Vos et al. [28], who found that higher levels of leisure-time PA is associated with higher scores in this domain for men. In the research by Yasunaga et al. [27] participants were classified according to quartiles of the PA level (1° - 4° and less active - more active, according to the caloric expenditure), verifying that men classified in the highest quartile had higher physical health scores.

In the present study, it was also found that moderate and vigorous PA intensities were associated with the physical health domain. Men who perform 300 min/week or more or of moderate activity and between 75-149 min/week of vigorous intensity PA had higher scores in the physical health domain than men who do not practice any activity. This association was also found by Lobo et al. [29]. Related to vigorous PA intensity, the findings of this study agree with the findings of Van den Berg et al. [30], which also found that men who reach the recommendation of vigorous PA intensity showed higher scores in the physical domain compared to those who do not.

For women, the level and intensities of PA were not associated with the mental health domain. This result differs from most of the literature [26, 31, 32]. However, Morimoto et al. [25] found that women who performed vigorous PA intensity (9 METS) had higher HRQL scores in almost all domains, except for social function and mental health. The lack of association between PA and mental health domain can be related to different categorizations of PA, different instruments to assess HRQL and evaluation of different age groups. Furthermore, Reid et al [33] have showed a connection among mental health, self-esteem and body image perceptions. Active women who did not achieve the desired body image did not improve self-esteem [33]. These influences may have played a role in the present results.

There was a positive association between moderate intensity and total activity leisure-time PA with mental health in men. Men who performed 150 min/week or more of leisure-time PA and a total of 300 min/week or more of moderate PA had higher scores in the mental health domain when compared with men who did not perform any PA. These findings are in agreement with the results of Lobo et al. [29], but differ from the findings of Van den Berg [30]. They evaluated the HRQL of men workers through the SF-12, classified as individuals who reach the recommendation for moderate or vigorous PA. It was concluded that only vigorous PA intensity was related to higher scores of HRQL in mental and physical domains. There was no association between HRQL with the recommended levels of moderate PA intensity, after adjustment for age, gender and psychosocial work factors, lifestyle, body mass index and oxygen consumption [30]. The difference from the present results may be due to use of different instruments to measure HRQL. The SF-36 was used in the present study and by Lobo et al. [29], whereas Van den Berg et al. [30] used the SF-12.

The level and intensities of PA were not associated with overall HRQL in women. This result differ from results from various studies [26–28, 32, 34, 35] who found that higher levels of PA are associated with higher scores on HRQL for women. However, in the study of Valadares et al. [36] this association was reversed, that means, women with higher levels of leisure-time PA had lower scores on HRQL. From these studies it is plausible to assume that the association between HRQL and the level and intensities of PA is influenced by different factors, such as the instruments and the form used to assess HRQL, age, cut-off level of PA and questionnaires to assess the PA levels. Thus, caution is suggested when comparing HRQL and PA levels.

For men, moderate and vigorous, and total leisure-time PA intensities were positively associated with higher HRQL scores. These results are in agreement with several studies [26–28, 33, 37] demonstrating that higher levels of leisure-time PA are associated with higher scores on the HRQL. Furthermore, Vuillemin et al. [14] demonstrated that men who perform more vigorous PA intensity had higher scores on HRQL than men who do not perform any activity.

This study employed a rigorous sampling selection which yielded in a representative sample of adults living in Rio Claro. Moreover, valid and widely used instruments during data collection were utilized. In addition, a control procedure to check for the quality of the data collected was also employed. This could be considered strengths of this study. However, although important, our findings should be carefully interpreted due to some limitations. This study was not adjusted by other factors that may have influence on HRQL (psychosocial work factors, lifestyle, body mass index, race, living with partner), and not included analysis of other HRQL domains (environmental and social). Future studies should explore the association between all four PA domains (leisure-time, occupational, transportation and household) according to the intensity of PA (moderate and vigorous) with the HRQL domains, stratified by gender.

The present study adds information on the literature on HRQL regarding intensities of PA. The results showed that the association of PA with the HRQL domains is distinct between the PA intensities and gender. Based on these findings, more assertive and effective interventions for the promotion of HRQL according to the PA intensities and gender may be developed. This represents an important step towards potential interventions and strategies with a broader impact used by health agencies.

## Conclusion and implications

It is concluded that physical and mental domains and overall HRQL are differently associated with different levels and intensities (moderate and vigorous) of leisure-time PA. Additionally, these associations are different for men and women depending on the HRQL domain being assessed. These findings complement the current literature and reinforce the importance of using statistical analyses that are stratified by gender, as well as the dose-response relationships.

## Abbreviations

HRQL: Health related to quality of life
IPAQ: International Physical Activity Questionnaire
SF 36: Medical Outcome Study 36- Item Short Form Health Survey.
